# Supplementary material for: Universal scaling laws of keyhole stability and porosity in 3D printing of metals
Source: Nat Commun. 2021 Apr 22;12:2379. doi: 10.1038/s41467-021-22704-0 (PMC8062476; doi:10.1038/s41467-021-22704-0)
Supplement: Supplementary file 1 — Supplementary Information [file 41467_2021_22704_MOESM1_ESM.pdf]

Supplementary Information for

Universal scaling laws of keyhole stability and porosity in 3D printing of metals

Zhengtao Gan, Orion L. Kafka, Niranjan Parab, Cang Zhao, Lichao Fang, Olle Heinonen, Tao Sun, Wing Kam Liu.

Correspondence to: zhengtao.gan@northwestern.edu, w-liu@northwestern.edu.

This PDF file includes:

Supplementary Methods 1 to 3
Supplementary Figs. 1 to 10
Supplementary Tables 1 to 3

Other Supplementary Files for this manuscript include the following:

Supplementary Movies 1 to 11
Supplementary Data 1 to 2

Supplementary Method 1

Multiphysics modeling of keyhole-mode laser melting

A multiphysics model is developed to capture the complex physical phenomena in keyhole-mode laser melting, including multiple reflections of the laser beam, heat and mass transfer, Marangoni flow, vaporization-induced recoil pressure, and transport of metal vapor plume. In the computational implementation, the computational domain, including a gas domain and a substrate domain, is compared with the x-ray experimental configuration in Supplementary Fig. 3. The volume fractions of gas and substrate phases are defined as α_g and α_l, respectively, and they sum to unity

$$\alpha_l + \alpha_g = 1 \tag{1}$$

The volume fraction equations for gas and substrate phases can be written as

$$\frac{\partial \alpha_g}{\partial t} + \nabla \cdot (\mathbf{u}\alpha_g) = \frac{\dot{m}\nabla\alpha_g}{\rho_g} \tag{2}$$

$$\frac{\partial \alpha_l}{\partial t} + \nabla \cdot (\mathbf{u}\alpha_l) = -\frac{\dot{m}\nabla\alpha_l}{\rho_l} \tag{3}$$

where t is the time, $\mathbf{u}$ is the velocity, $\dot{m}$ is the mass transfer rate due to vaporization, and ρ_g denotes the density of the gas phase and ρ_l denotes the density of the substrate phase, such that the total density ρ at each point $\rho = \rho_g\alpha_g + \rho_l\alpha_l$. The unit normal vector $\mathbf{n}$ and curvature κ of the interface Γ can be calculated from the volume faction:

$$\mathbf{n} = \frac{\nabla\alpha_l}{|\nabla\alpha_l|} = \frac{-\nabla\alpha_g}{|\nabla\alpha_g|} \tag{4}$$

$$\kappa = -\nabla \cdot \mathbf{n} \tag{5}$$

Momentum conservation is governed by the Naiver-Stokes equation:

$$\frac{\partial}{\partial t}(\rho\mathbf{u}) + \mathbf{u}\cdot\nabla(\rho\mathbf{u}) = -\nabla p + \nabla\cdot[\mu(\nabla\mathbf{u} + \nabla\mathbf{u}^T)] + \mathbf{f_v} + \mathbf{f_s} \tag{6}$$

where p is the pressure, μ is the dynamic viscosity, superscript T indicates the transpose, and $\mathbf{f_v}$ and $\mathbf{f_s}$ denote the volumetric force and the continuum surface force (CSF) discretization of interfacial forces acting over the interface Γ between substrate and gas. The $\mathbf{f_v}$ includes gravity $\mathbf{f_{gravity}}$ and Darcy force $\mathbf{f_{Darcy}}$:

$$\mathbf{f_v} = \mathbf{f_{gravity}} + \mathbf{f_{Darcy}} = \rho\mathbf{g} - \frac{A_{\mathrm{mush}}(1-f_{\mathrm{liq}})^2}{(f_{\mathrm{liq}}{}^3 + B)}\mathbf{u}\alpha_l \tag{7}$$

where $\mathbf{g}$ is the gravitational acceleration, A_{mush} is the mushy zone constant, B is a small number to prevent division by zero, and f_{liq} is the liquid fraction that can be calculated from the temperature (*1*).

The interfacial forces include the surface tension force $\mathbf{f_{ST}}$, the Marangoni force $\mathbf{f_{Marangoni}}$, and the recoil force due to vaporization $\mathbf{f_{recoil}}$:

$$\mathbf{f_s} = \mathbf{f_{ST}} + \mathbf{f_{Marangoni}} + \mathbf{f_{recoil}} \tag{8}$$

Those forces can be expressed as

$$\mathbf{f_{ST}} = \sigma\kappa\nabla\alpha_l \tag{9}$$

$$\mathbf{f_{Marangoni}} = \frac{\mathrm{d}\sigma}{\mathrm{d}T}[\nabla T - \mathbf{n}(\mathbf{n}\cdot\nabla T)]|\nabla\alpha_l|(\frac{2\rho}{\rho_l + \rho_g}) \tag{10}$$

$$\mathbf{f_{recoil}} = (P_{\mathrm{recoil}} - P_{\mathrm{atm}})\nabla\alpha_l \tag{11}$$

where σ is the surface tension coefficient, T is the temperature, $\frac{\mathrm{d}\sigma}{\mathrm{d}T}$ is the temperature coefficient of surface tension defined as the partial derivative of the surface tension coefficient with respect to temperature, P_{recoil} is the vaporization-induced recoil pressure, and P_{atm} is the atmospheric pressure.

To approximate the mass transfer rate $\dot{m}$ and the recoil pressure P_{recoil} across the Knudsen layer, the Hertz-Langmuir relation (*2*) is typically used. Since the Hertz-Langmuir relation is only valid at high vaporization intensities (when temperature is much higher than boiling point or in vacuum), the $\dot{m}$ is calculated by bridging the vaporization regimes with a smoothed third-order polynomial:

$$\dot{m} = \begin{cases} 0, & 0 \le T \le T_L \\ a_1 T^3 + b_1 T^2 + c_1 T + d_1, & T_L \le T \le T_H \\ (1-\beta_R)\sqrt{\frac{M_l}{2\pi R T}} P_{\mathrm{sat}}, & T_H \le T \le +\infty \end{cases} \quad (12)$$

where a_1, b_1, c_1, and d_1 are fitting coefficients, β_R is the retro-diffusion coefficient, M_l is the molar mass of the vaporized species, R is the gas constant, and the temperature thresholds T_L and T_H represent the low and high vaporization intensity regimes. The temperature-dependent saturated vapor pressure P_{sat} is calculated with the Clausius-Clapeyron law (*3*) as

$$P_{\mathrm{sat}} = P_{\mathrm{atm}} \exp\left[\frac{M_l L_v}{R T_v}\left(1 - \frac{T_v}{T}\right)\right] \quad (13)$$

where L_v is the latent heat of vaporization, and T_v is the boiling point at atmospheric pressure.

To consider the effects of the atmospheric pressure, the recoil pressure can be expressed as

$$P_{\mathrm{recoil}} = \begin{cases} P_{\mathrm{atm}}, & 0 \le T \le T_L \\ a_2 T^3 + b_2 T^2 + c_2 T + d_2, & T_L \le T \le T_H \\ \frac{1}{2}(1+\beta_R) P_{\mathrm{sat}}, & T_H \le T \le +\infty \end{cases} \quad (14)$$

where a_2, b_2, c_2, and d_2 are fitting coefficients.

Energy conservation equation can be written as

$$\frac{\partial}{\partial t}(\rho H) + \nabla \cdot (\rho \mathbf{u} H) = \nabla \cdot (k \nabla T) + Q_s \quad (15)$$

$$H = h_{\mathrm{ref}} + \int_{T_{\mathrm{ref}}}^{T} c_p dT + f_{\mathrm{liq}} L_m \quad (16)$$

where H is the enthalpy of the material, k is the thermal conductivity, h_{ref} is the reference enthalpy with respective to the reference temperature T_{ref}, c_p is the heat capacity, and L_m is the latent heat of melting. The volumetric source term Q_s represents the energy source applied on the interface between substrate and gas Γ, which includes radiative source Q_{rad}, laser source Q_{laser}, and evaporative source Q_{evp}:

$$Q_s = Q_{\mathrm{rad}} + Q_{\mathrm{laser}} + Q_{\mathrm{evp}} \quad (17)$$

Those sources can be expressed as

$$Q_{\mathrm{rad}} = -\sigma_{\mathrm{SB}} \varepsilon (T^4 - T_\infty^4) |\nabla \alpha_l| (\frac{2\rho}{\rho_l + \rho_g}) \quad (18)$$

$$Q_{\mathrm{laser}} = q_{\mathrm{laser}} |\nabla \alpha_l| (\frac{2\rho}{\rho_l + \rho_g}) \quad (19)$$

$$Q_{\mathrm{evp}} = -L_v \dot{m} |\nabla \alpha_l| (\frac{2\rho}{\rho_l + \rho_g}) \quad (20)$$

where σ_{SB} is the Stefan-Boltzmann constant, ε is the material emissivity, T_∞ is the ambient temperature. To account for the multiple reflection absorptions of laser beam, the absorbed energy flux q_{laser} is calculated using a ray tracing method (*4*,*5*):

$$q_{\text{laser}} = I_0(r)(\mathbf{I_0} \cdot \mathbf{n_0})\alpha_{\text{Fr}}(\theta_0) + \sum_{m=1}^{N} I_m(r,z)(\mathbf{I_m} \cdot \mathbf{n_m})\alpha_{\text{Fr}}(\theta_m) \tag{21}$$

$$\alpha_{\text{Fr}}(\theta) = 1 - \frac{1}{2}\left[\frac{1+(1-\alpha_0\cos\theta)^2}{1+(1+\alpha_0\cos\theta)^2} + \frac{\alpha_0^2-2\alpha_0\cos\theta+2\cos^2\theta}{\alpha_0^2+2\alpha_0\cos\theta+2\cos^2\theta}\right] \tag{22}$$

$$I_0(r) = \frac{2P}{r_0^2}\exp\left[-\frac{2(r-V_s t)^2}{r_0^2}\right] \tag{23}$$

where I represents the laser energy flux, $\mathbf{I}$ represents the unit vector of the beam, θ represents the angle between the laser beam and normal of the keyhole interface Γ, $\mathbf{n}$ is the unit normal vector of the interface Γ. The subscript 0 denotes the incident beam and m denotes the mth reflections. The Fresnel absorption coefficient α_{Fr} is applied, and α_0 is a coefficient related to the types of lasers and materials, P is the laser power, r_0 is the laser spot radius, V_s is the scan speed, r is the radial coordinate, and z is the vertical z-coordinate. More detailed calculation of the ray tracing method and its validation are provided elsewhere (*6*). An illustrative result of the ray tracing is shown in Supplementary Fig. 4.

A conservation equation for metal vapor concentration in the gas phase is coupled with the momentum conservation equation:

$$\frac{\partial}{\partial t}(\rho_g\alpha_g Y_l) + \nabla\cdot(\mathbf{u}\rho_g\alpha_g Y_l) = \nabla\cdot(\alpha_g\rho_g D\nabla Y_l) + \dot{m}|\nabla\alpha_l| \tag{24}$$

where Y_l represents the mass fraction of substrate species (one major component in the substrate is considered in this study) and D is the mass diffusion coefficient. The gas density is defined using the ideal gas law for an incompressible flow:

$$\rho_g = \frac{p_{op[M_l Y_l + M_g(1-Y_l)]}}{RT} \tag{25}$$

where p_{op} is the operating pressure in the experimental chamber, M_g is the molar mass of the gas (e.g., argon), and M_s is the molar mass of the substrate (e.g., aluminum) species.

External boundaries of computational domain are assumed to be adiabatic since they are sufficiently far from the heat source and the processing time is sufficiently short. A no slip condition for momentum equations and a zero diffusive flux for the species equation are set on the external boundaries except the top boundary, which is set as a fluid outlet with ambient pressure and zero mass fraction of the substrate species.

The governing equations are solved by the finite volume method using the non-iterative PISO scheme within ANSYS FLUENT 2020 R1 (*7*) using user-defined functions (UDFs). The Second Order Implicit Scheme is used for the transient formulation. The Least Squared Cell-Based scheme is used to compute gradients, PRESTO is used to compute pressures, and Second Order Upwind is used for momentum spatial discretization. An explicit Volume of Fluid (VOF) solver is applied with the CICSAM discretization scheme (*8*). The energy equation is discretized using the Power Law scheme, and the species equation is discretized using the Second Order Upwind scheme. An automatic local grid refinement technique is used. A uniform hexahedral mesh with an edge length of 8 μm is used initially, and then two levels of local refinement (mesh edge length down to 2 μm) is specified when the temperature associated with the region is higher than the solidus temperature of the material. A variable time step ranging from 1×10^{-10} s to 1×10^{-8} s is used such that the global Courant number is smaller than one.

Thirty simulations with different laser power and scan speed are conducted (ten for the Al6061 substate and twenty for the Ti-6Al-4V substrate). The thermophysical properties are provided in Supplementary Table 1, and the computational parameters are given in Supplementary Table 2. Quantitative comparisons of keyhole aspect ratio and melt pool size between x-ray experiments and multiphysics simulations are presented in Supplementary Figs. 5 and 6.

Supplementary Method 2

Energy balance calculation and approximation

The energy balance in laser melting or additive manufacturing can be expressed as

$$E_{\mathrm{laser}} = E_{\mathrm{reflect}} + E_{\mathrm{convect}} + E_{\mathrm{radiate}} + E_{\mathrm{evaporiate}} + E_{\mathrm{spatter}} + E_{\mathrm{conduct}} \quad (26)$$

where E_{laser} is the total laser energy deposited during the process, E_{reflect} is the reflected energy, E_{convect}, is the convection energy losses, E_{radiate} is the radiation energy loss and E_{spatter} is the spattering energy loss. The portion of energy transferred within the substrate due to conduction is denoted by E_{conduct}.

A power balance can be obtained by deriving the energy balance equation with respect to time, such that a transient power balance or averaged power balance during a period of time can be analyzed:

$$P_{\mathrm{laser}} = P_{\mathrm{reflect}} + P_{\mathrm{convect}} + P_{\mathrm{radiate}} + P_{\mathrm{evaporate}} + P_{\mathrm{spatter}} + P_{\mathrm{conduct}} \quad (27)$$

where P_{laser} is equal to laser power P. Similarly, the laser power consists of power losses due to reflection P_{reflect}, convection P_{convect}, radiation P_{radiate}, evaporation $P_{\mathrm{evaporate}}$, spattering P_{spatter}, and transferred power due to conduction P_{conduct}.

Those powers can be approximated based on the multiphysics model:

$$P_{\mathrm{reflect}} = 1 - \iint_{\Gamma} q_{\mathrm{laser}}\, \mathrm{d}S \quad (28)$$

$$P_{\mathrm{convect}} = \iint_{\Gamma'} \rho_g c_{\mathrm{pg}} T\, \mathbf{u} \cdot \mathbf{n}_{\Gamma'}\, \mathrm{d}S \quad (29)$$

$$P_{\mathrm{radiate}} = \iint_{\Gamma} \sigma_{\mathrm{SB}} \varepsilon (T^4 - T_\infty^4)\, \mathrm{d}S \quad (30)$$

$$P_{\mathrm{evaporate}} = \iint_{\Gamma} L_v \dot{m}\, \mathrm{d}S \quad (31)$$

$$P_{\mathrm{conduct}} = P_{\mathrm{absorb}} - P_{\mathrm{convect}} - P_{\mathrm{radiate}} - P_{\mathrm{evaporate}} - P_{\mathrm{spatter}} \quad (32)$$

where the subscript g denotes the gas phase, Γ is the interface between substrate and gas, and Γ' is a flat surface at the top of the substrate. A schematic of those two interfaces is shown in Supplementary Fig. 7. Other parameters have been described previously. These integrals are computed at each time step after the keyhole depression completes a rapid growth. Mean and standard deviation of the integrals during the quasi-steady state are recorded. The power loss due to spattering P_{spatter} cannot be accurately calculated from the current model. Alternatively, the average power loss due to spattering $\bar{P}_{\mathrm{spatter}}$ can be approximated as

$$\bar{P}_{\mathrm{spatter}} = \frac{\mathrm{V}_{\mathrm{spatter}} \rho_l c_{\mathrm{pl}} T_v}{t_0} \quad (33)$$

where V_{spatter} is the volume of spattered droplets that can be approximated from the x-ray images by assuming that the separated droplets are spherical (an illustrative result is shown in Supplementary Fig. 8). The subscript l denotes the liquid phase, and t_0 is the observation time of x-ray imaging. Vaporization temperature T_v is used in the approximation because most of the

spattered droplets are ejected from the keyhole depression region where the temperature is near the T_v. Experimentally, average power due to conduction can be measured by attached thermocouples (*6*). Those experimental data are used to validate the predicted data from the model.

Supplementary Method 3

Mathematical derivation of scaling parameters

To correlate the keyhole size and aspect ratio with process parameters and material properties in a compact form, scaling laws are derived from normalized governing equations and boundary conditions with appropriate assumptions and simplifications. We begin with a heat conduction problem in a semi-infinite region without considering the powder layer, Marangoni flow, evaporation and spattering. Previous analysis of the energy balance has shown that energy loss due to evaporation and spattering can be ignored as comparison with heat conduction. We consider a well-developed keyhole or transition regime where the laser energy is high enough to melt the powder around the laser, and thus the effect of particle morphology on the keyhole is neglectable. Ultrahigh speed x-ray observation (*9*) and qualitative arguments (*10*) support this assumption. The effect of the Marangoni flow will be analyzed later. We neglect the latent heat of melting because it is much smaller than the energy required to heat material to the melting point and consequently only affects the temperature distribution near the mushy zone. We also assume temperature-independent thermophysical properties at the melting point in the derivation, a previous study indicates that the inclusion of the temperature-dependent thermophysical properties does not change the keyhole depth qualitatively (*11*).

We consider a laser beam scanning a substrate with the scan speed V_s along direction x. Direction y is the transverse coordinate, and z is the normal into the substrate surface. In a reference frame that moves with the laser beam at a fixed relative location, the temperature field is governed by

$$\rho C_p \left(\frac{\partial T}{\partial t} + \mathbf{u} \cdot \nabla T \right) = \nabla \cdot (k \nabla T) \tag{34}$$

$$\mathbf{u} = -V_s \cdot \mathbf{i} \tag{35}$$

where ρ is the density of the solid, C_p is the heat capacity, T is the temperature, $\mathbf{u}$ is the velocity of the reference frame, V_s is the speed of the frame which is equal to scan speed of the heat source, and $\mathbf{i}$ is the unit vector in the *x* direction. We substitute Equation 35 into Equation 34 and consider a steady state:

$$\frac{V_s}{\alpha} \frac{\partial T}{\partial x} + \nabla^2 T = 0 \tag{36}$$

where α is the thermal diffusivity that is defined as $\alpha = \frac{k}{\rho C_p}$.

The boundary condition considering a Gaussian heat source can be expressed as

$$\begin{cases} k \frac{\partial T}{\partial z} = \frac{2\eta P}{\pi r_0^2} \exp\left[\frac{2(x^2+y^2)}{r_0^2} \right], \ z = 0 \\ \qquad\qquad\qquad\qquad T = T_0, \ r \to \infty \end{cases} \tag{37}$$

where η is the effective laser absorptivity, P is the laser power, r_0 is the laser spot radius, T_0 is the preheat temperature, and r is defined as $r = \sqrt{x^2 + y^2 + z^2}$.

The goal of normalization is to acquire an equivalent but compact representation governed by a minimal set of dimensionless parameters. We define the dimensionless groups by dividing the temperature and spatial variables by their natural scaling factors, which are combinations of the dimensional parameters in this problem, as

$$x^* = \frac{x}{\delta_x} = \frac{x}{r_0} \tag{38}$$

$$y^* = \frac{y}{\delta_y} = \frac{y}{r_0} \tag{39}$$

$$z^* = \frac{z}{\delta_z} = z\sqrt{\frac{V_s}{\alpha r_0}} \tag{40}$$

$$T^* = \frac{T - T_0}{T_s} = \frac{(T - T_0)\pi\rho C_p\sqrt{\alpha V_s r_0^3}}{\eta P} \tag{41}$$

where superscript $*$ indicates the dimensionless parameter, δ_x, δ_y, and δ_z are the length scales in x, y, and z, respectively, and we define $\delta_x = \delta_y = r_0$ and $\delta_z = \sqrt{\frac{\alpha r_0}{V_s}}$. The thermal scale T_s is defined as $T_s = \frac{\eta P}{\pi\rho C_p\sqrt{\alpha V_s r_0^3}}$ so that the normalized governing equation and boundary conditions have the most compact form possible, given by

$$\frac{V_s r_0}{\alpha}\frac{\partial T^*}{\partial x^*} + \nabla^{*2}T^* = 0 \tag{42}$$

$$\begin{cases} \frac{\partial T^*}{\partial z^*} = \exp[2(x^{*2} + y^{*2})], \; z^* = 0 \\ T^* = 0, \; r^* \to \infty \end{cases} \tag{43}$$

Based on the normalized Equations (42) and (43), we define $p = \frac{\alpha}{V_s r_0}$ and the dimensionless temperature, which only depends on dimensionless coordinates and p:

$$T^* = f(x^*, y^*, z^*, p) \tag{44}$$

where the dimensionless parameter p represents the ratio between the thermal transfer speed $\frac{\alpha}{r_0}$ and scan speed V_s. The form of the function f cannot be obtained by using dimensional alone.

We are also interested in the size of a specific isotherm, for example liquidus temperature T_l, so we define

$$T_l^* = \frac{(T_l - T_0)\pi\rho C_p\sqrt{\alpha V_s r_0^3}}{\eta P} \tag{45}$$

The maximum depth of the melting isotherm z_m^* is determined by the relation

$$T_l^* = g(x^*, y^*, z^*, p) \tag{46}$$

where $y^* = 0$ and the position of the maximum depth can be found from the condition

$$\frac{\partial g}{\partial x} = 0 \tag{47}$$

Thus, the melt depth z_m^* is a function of T_l^* and p only

$$z_m^* = s(T_l^*, p) = s\left(\frac{(T_l - T_0)\pi\rho C_p\sqrt{\alpha V_s r_0^3}}{\eta P}, \frac{\alpha}{V_s r_0}\right) \tag{48}$$

In the keyhole mode or transition mode, the keyhole depth e is approximately equal to the melt pool depth z_m. The forms of the functions g and s cannot be obtained by using dimensional alone. The usage of the different symbols implies that the forms of those functions are different. Thus, the normalized keyhole depth is scaled by

$$e_z{}^* = \frac{e}{\delta_z} = \frac{e}{\sqrt{\frac{\alpha r_0}{V_s}}} = s'(T_l^*, p) = s'(\frac{\eta P}{(T_l - T_0)\pi\rho C_p\sqrt{\alpha V_s r_0^3}}, \frac{\alpha}{V_s r_0}) \quad (49)$$

$$\text{Thermal diffusion length } \delta_z = \sqrt{\frac{\alpha r_0}{V_s}} \quad (50)$$

$$\text{Normalized enthalpy enth}^* = \frac{P}{(T_l - T_0)\pi\rho C_p\sqrt{\alpha V_s r_0^3}} \quad (51)$$

$$\text{Normalized diffusion length } L_d^* = \frac{\delta_z}{r_0} = \sqrt{\frac{\alpha}{V_s r_0}} \quad (52)$$

Thus, based on the above dimensional analysis the keyhole depth e normalized by thermal diffusion length δ_z is a universal function of the normalized enthalpy enth^*, normalized diffusion length L_d^*, and the absorptivity η. We found a linear relationship between $e_z{}^*$ and the ratio of ηenth^* and L_d^* as

$$e_z{}^* \propto \frac{\eta\text{enth}^*}{L_d^*} \quad (53)$$

$$e^* = \frac{e}{r_0} = e_z{}^* L_d^* \propto \eta\text{enth}^* \quad (54)$$

The effects of liquid metal flow on the melt pool dynamics are described by a set of dimensionless numbers:

$$\text{Ra} = \frac{\rho g\beta(T_l - T_0)r_0^3}{\mu\alpha} \quad (55)$$

$$\text{Ma} = \left|\frac{\text{d}\sigma}{\text{d}T}\right|\frac{(h_l - h_0)r_0}{C_p\mu\alpha} \quad (56)$$

$$\text{Pr} = \frac{\mu}{\rho\alpha} \quad (57)$$

$$\text{We} = \frac{\rho U_\sigma^2 r_0}{\sigma} \quad (58)$$

$$\text{Bo} = \frac{\rho g r_0^2}{\sigma} \quad (59)$$

$$\text{Ca} = \frac{\mu U_\sigma}{\sigma} \quad (60)$$

$$U_\sigma = \left|\frac{\text{d}\sigma}{\text{d}T}\right|\frac{(h_l - h_0)}{C_p\mu} \quad (61)$$

where Rayleigh number Ra is associated with buoyancy-driven flow, Marangoni number Ma characterizes the Marangoni flow, and Prandtl number Pr affects the morphology of the melt pool driven by Marangoni flow (*12*). The Weber number We, Bond number Bo and Capillary number Ca affect the shape of the melt pool free surface (*12*). Supplementary Table 3 lists the values of those dimensionless numbers for three substrate materials investigated in this study: Al6061, Ti6Al4V, and SS316. Those dimensionless numbers are roughly on the same order of magnitude, implying that fluid flow is similar for different materials under the same process conditions. Thus, ignoring the parameters associated with fluid flow in the melt pool ought not significantly affect the scaling for keyhole depth or aspect ratio.

Based on a procedure similar to those discussed above, scaling laws for the front angle and inlet length of keyhole can also be obtained as shown in Supplementary Fig. 9 (Data S1). The

keyhole front angle is approximately proportional to the keyhole aspect ratio, and thus it correlates well with the Keyhole number. We also conclude that the normalized keyhole inlet length $l^* = \frac{l}{r_0}$ is dominated by the normalized diffusion length L_d^*.

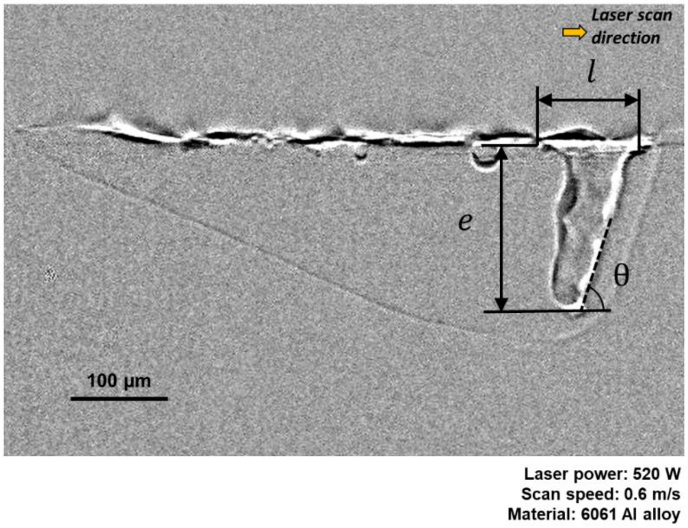

Supplementary Fig. 1. Representative x-ray image of the melt pool and keyhole depression after the processing steps described have been conducted. Keyhole depth e, inlet length l, and front angle θ are marked in the image.

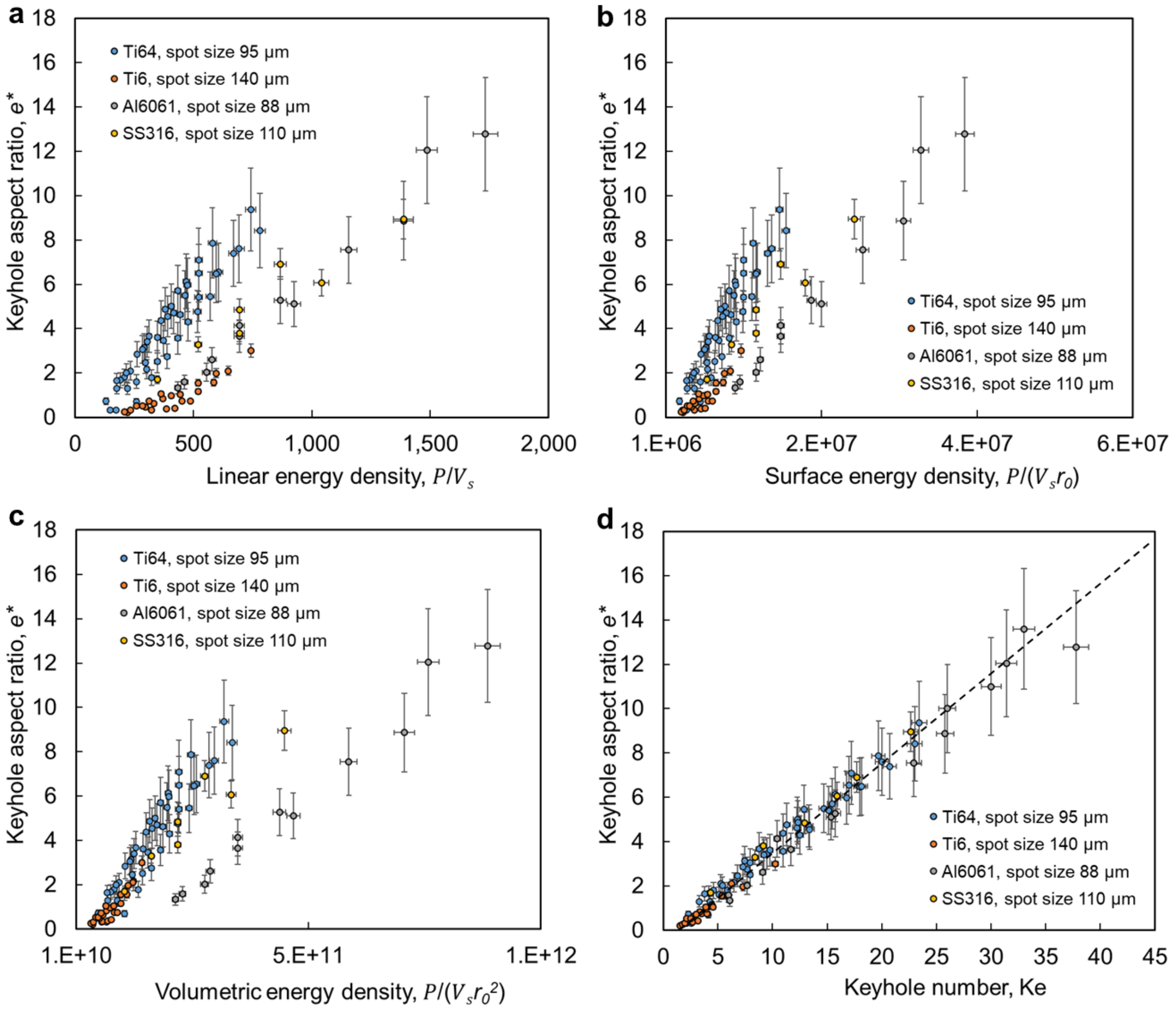

Supplementary Fig. 2. Correlation between keyhole aspect ratio and different descriptors. The maximum and minimum of keyhole aspect ratio during the time period when the laser scans 2 mm length at the middle of the sample are marked as vertical error bars. Horizonal error bars indicate 3% error amount to account for uncertainties of the process parameters and material properties. (**a**) Linear energy density. (**b**) Surface energy density. (**c**) Volumetric energy density. (**d**) Keyhole number. All the data collapse to a single curve when the Keyhole number is used as descriptor.

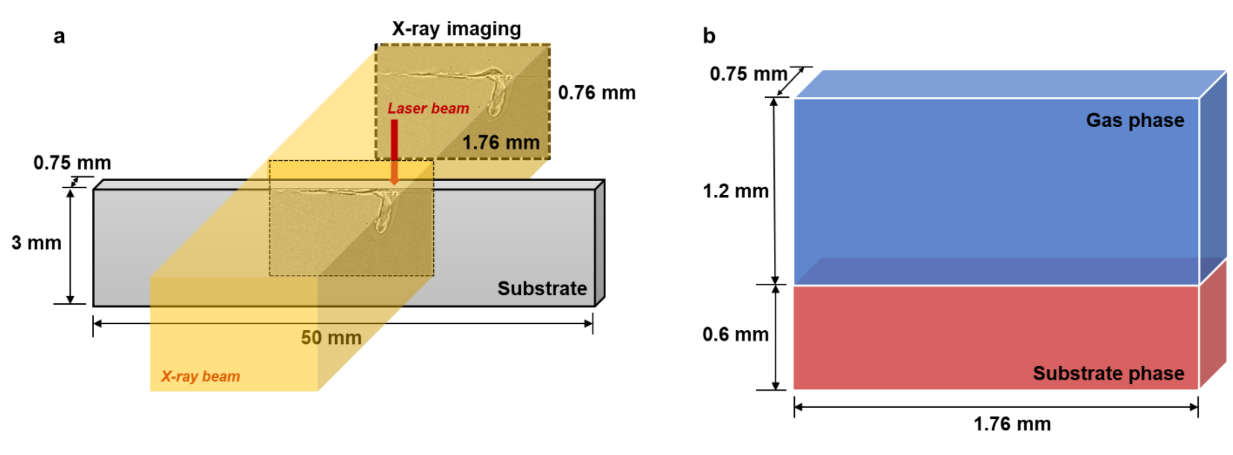

Supplementary Fig. 3. X-ray experimental configuration and computational domain. (**a**) Size of sample and x-ray imaging region. (**b**) Computational domain size.

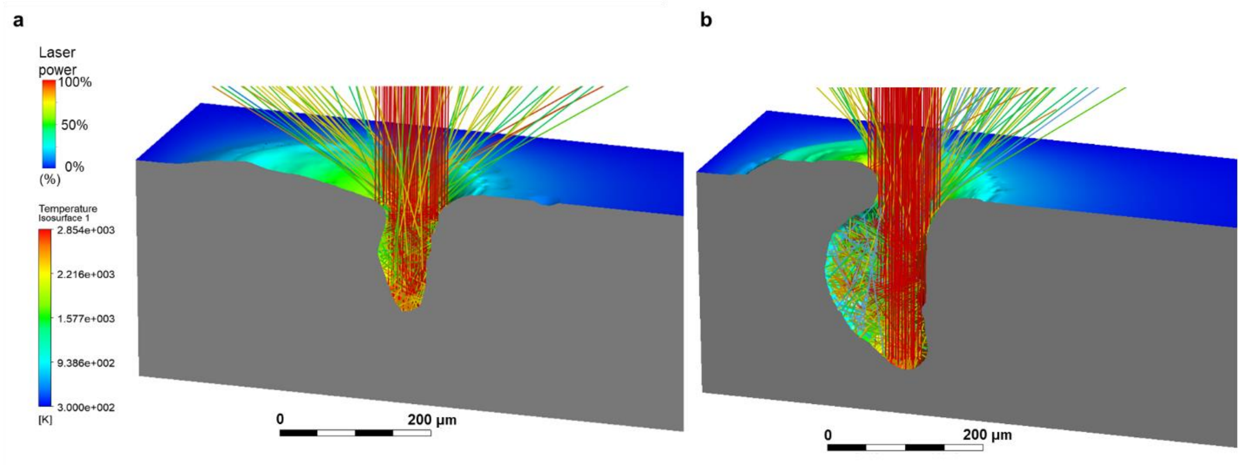

Supplementary Fig. 4. An illustrative result of the ray tracing method. Half of the domain is shown. The laser rays are colored by relatively power. The incoming rays arrive at 100% power and lose energy upon reflection. (**a**) The case with laser power 416 W, scan speed 0.6 m/s, and material Al6061. (**b**) The case with laser power 520 W, scan speed 0.45 m/s, and material Al6061.

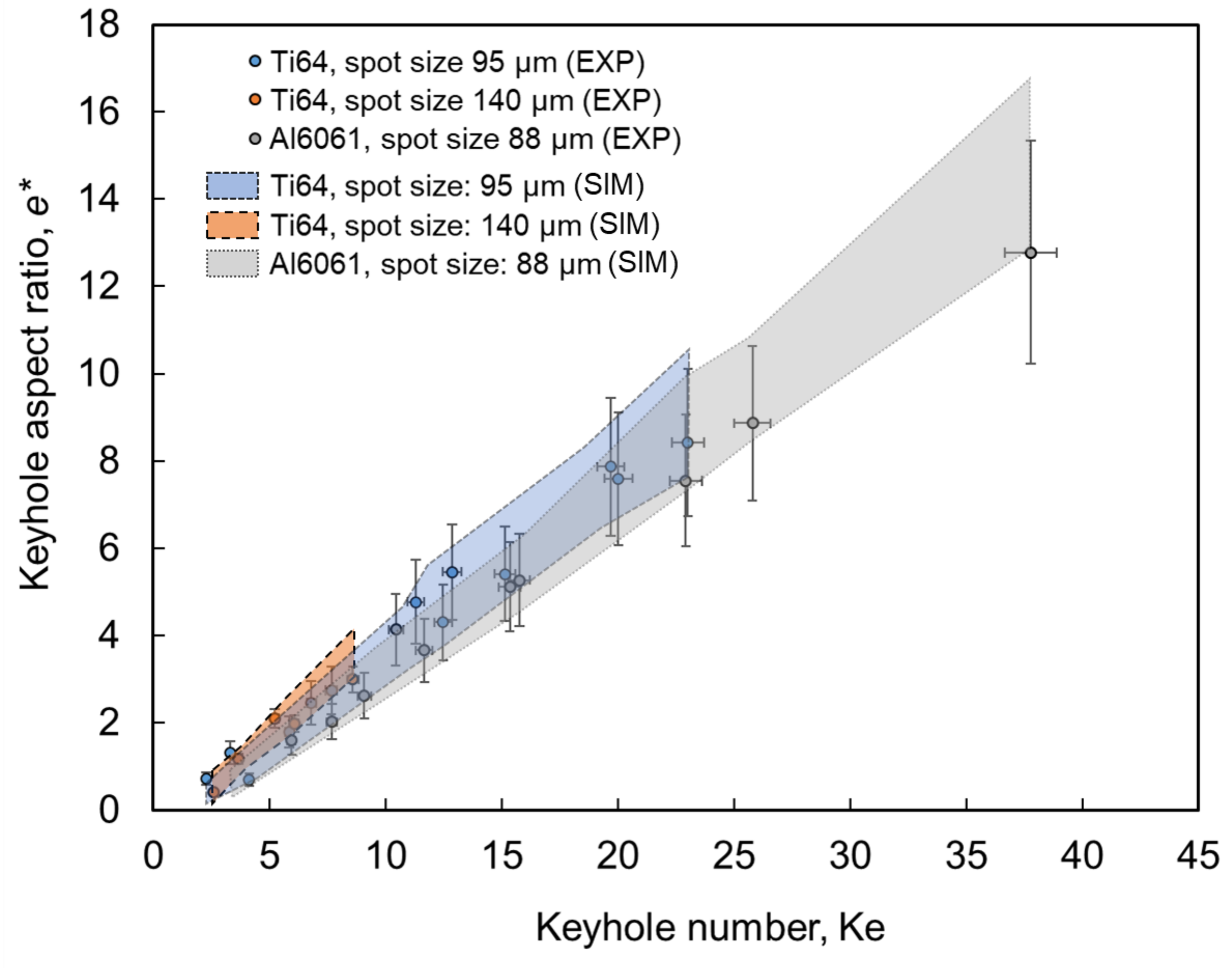

Supplementary Fig. 5. Comparison between experimental (EXP) and simulation (SIM) keyhole aspect ratios with different process parameters and materials. The error bars indicate the maximum and minimum of keyhole aspect ratio during the time period when the simulated process reaches a quasi-steady state.

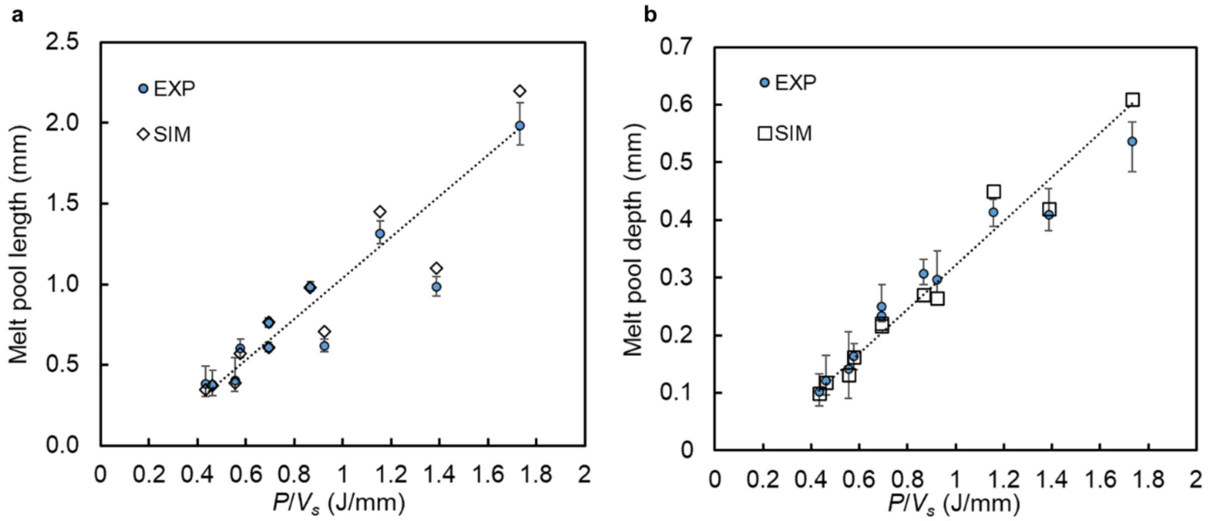

Supplementary Fig. 6. Comparison between experimental (EXP) and simulation (SIM) melt pool sizes for aluminum alloy Al6061. The length of the melt pool was quantified in the x-ray image by measuring the distance between the front end and the tail end of the fusion boundary in the laser scan direction. The length can also be quantified in the simulation results based on the location of the isotherm of the solidus temperature of the material. The depth of the melt pool can be quantified based on a similar metric. The error bars indicate the maximum and minimum of the melt pool length or depth during the time period when the simulated process reaches a quasi-steady state. (**a**) melt pool length. (**b**) melt pool depth.

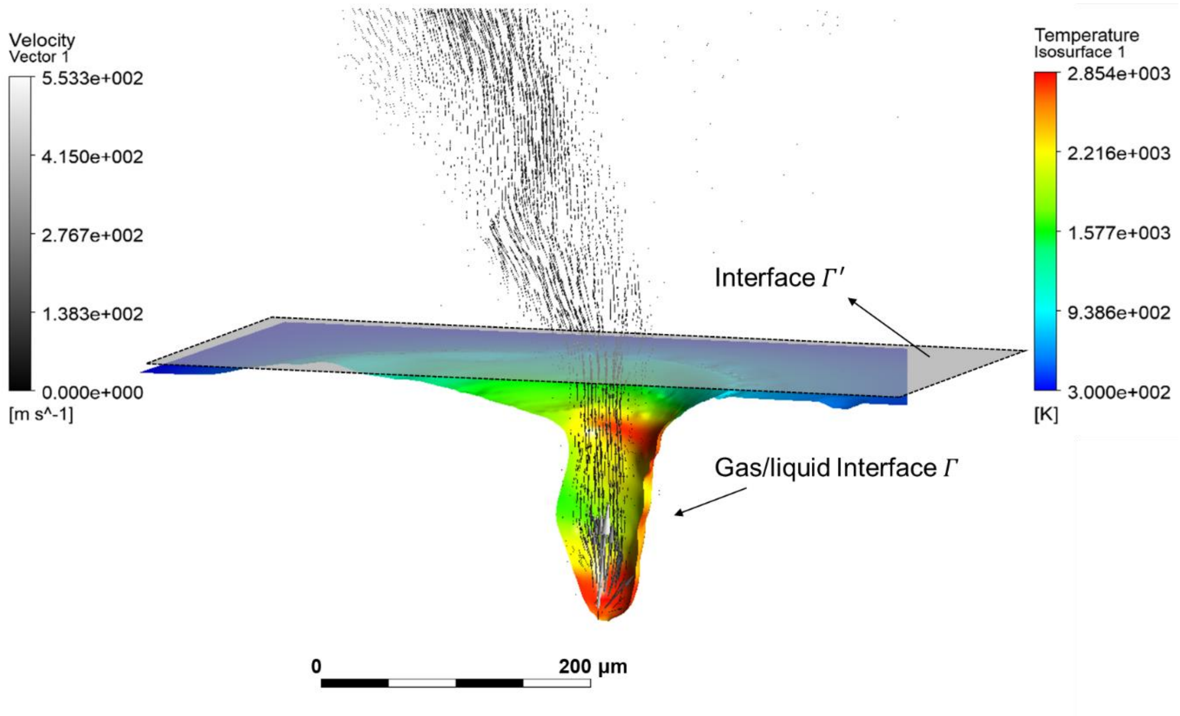

Supplementary Fig. 7. A schematic of the two interfaces used to compute the energy balance. Vapor plume velocity is showed as an arrow field.

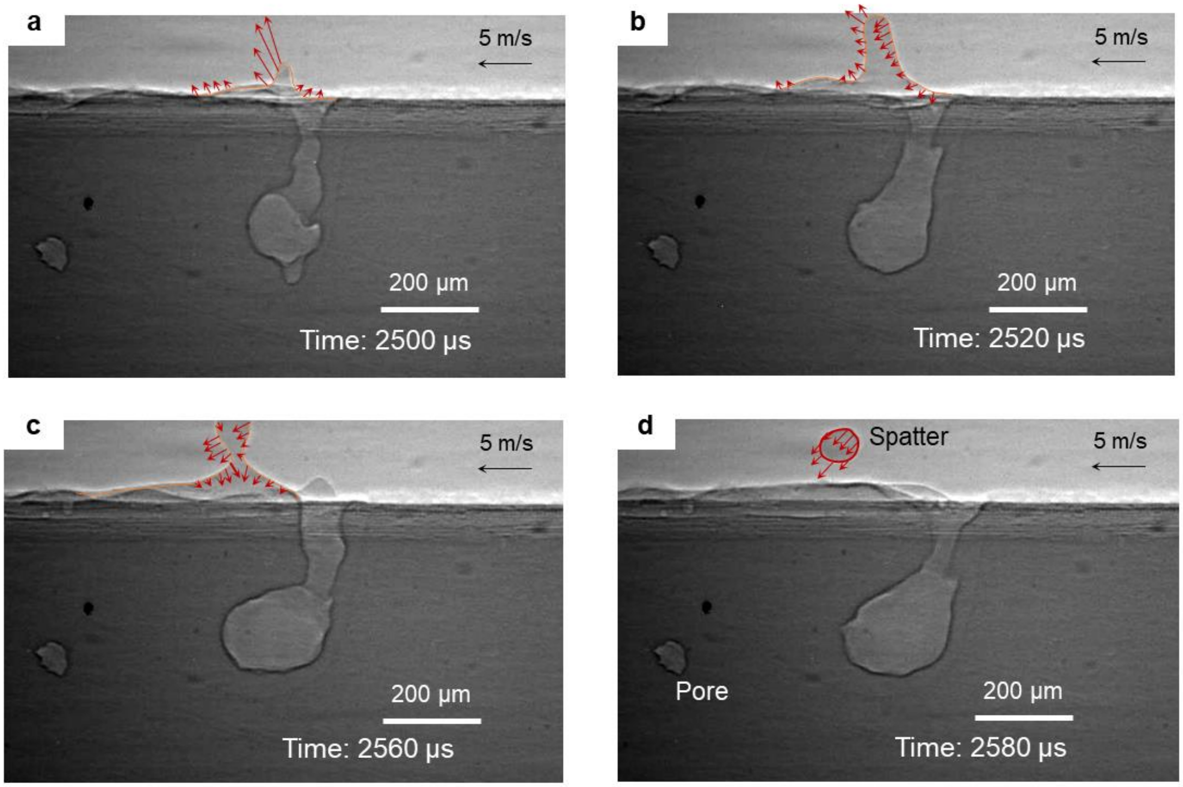

Supplementary Fig. 8. X-ray image series at different time showing the spatter formation and evolution in laser melting of Al6061. The red arrows show the velocity vector of the interface of the spatter droplet. A reference velocity, 5 m/s, is given. (**a**) 2500 μs. (**b**) 2520 μs. (**c**) 2560 μs. (**d**) 2580 μs.

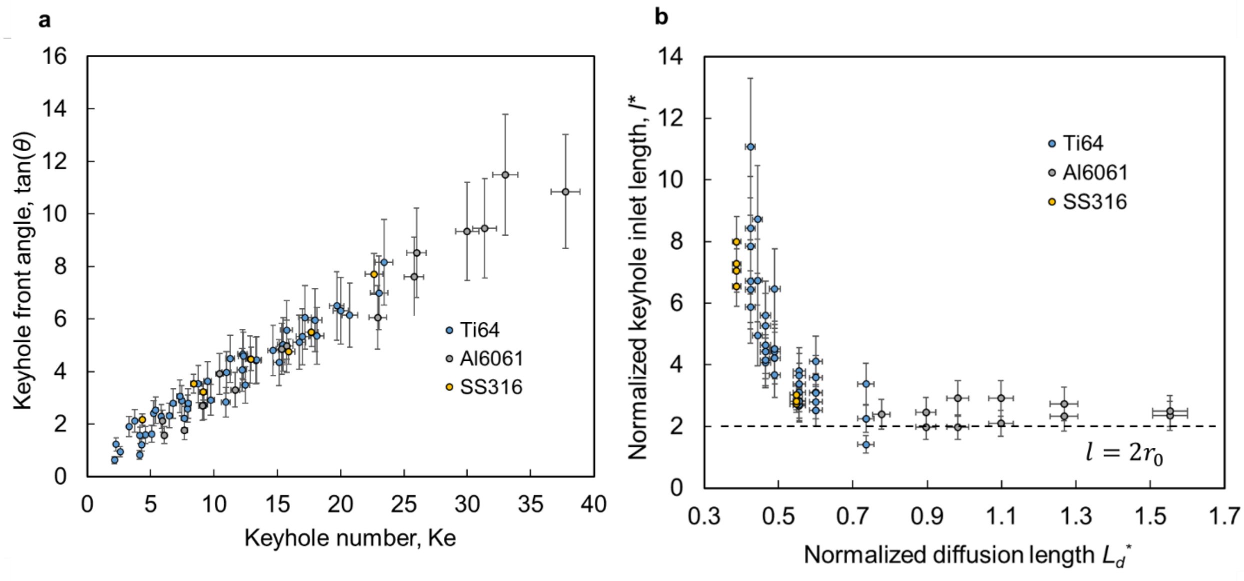

Supplementary Fig. 9. Scaling relations for the front angle and inlet length of a keyhole. The maximum and minimum of keyhole front angle or inlet length during the time period when the laser scans 2 mm length at the middle of the sample are marked as vertical error bars. Horizonal error bars indicate 3% error amount to account for uncertainties of the process parameters and material properties. (**a**) Tangent of the keyhole front angle. (**b**) Keyhole inlet length. A horizontal line denotes where the keyhole inlet length equals the laser beam diameter.

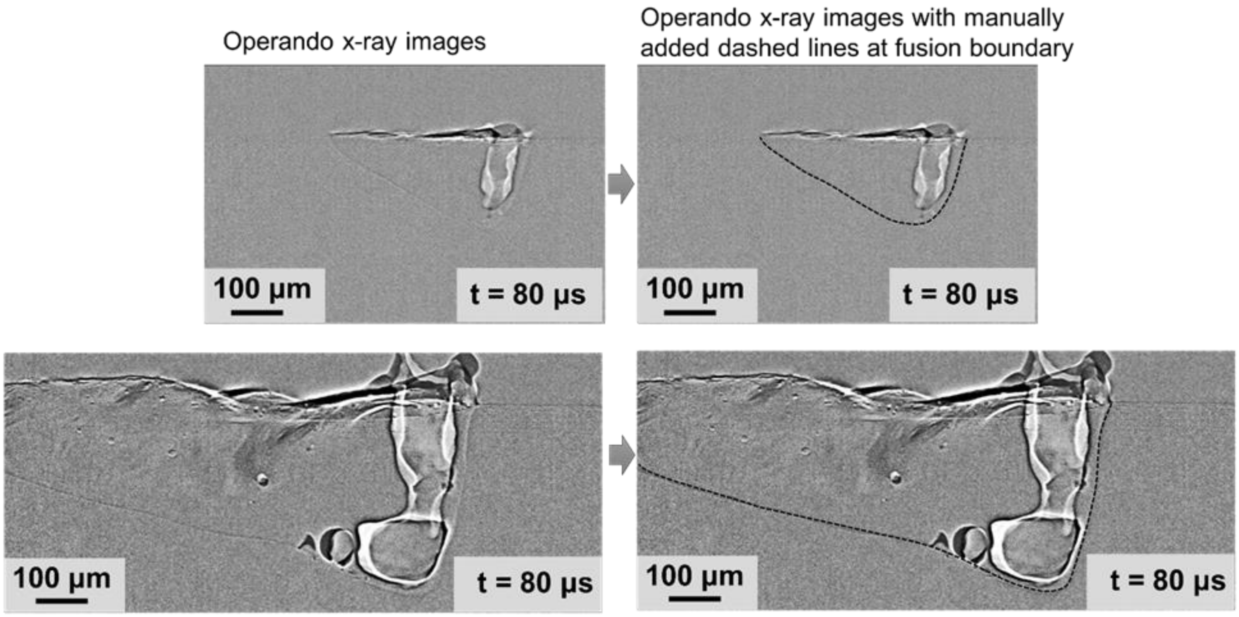

Supplementary Fig. 10. Comparison between operando x-ray image and the same image with manually added dashed line at the fusion boundary. This dashed line emphasizes the fusion boundary, which is otherwise observable as a faint line that may be obfuscated in print.

Properties & units	Ti64	Al6061	SS316
Density ρ_l $(\frac{kg}{m^3})$	4420, T < 1933 K<br>3920, T ≥ 1933 K	2705, T < 915 K<br>2415, T ≥ 915 K	7950, T < 1723 K<br>6881, T ≥ 1723 K
Heat capacity (liquid) C_{pl} $(\frac{J}{kg \cdot K})$	0.1734T+452.72, T < 1933 K<br>830, T ≥ 1933 K	0.486T+725, T < 915 K<br>1170, T ≥ 915 K	0.24T+420.8, T < 1723 K<br>790, T ≥ 1723 K
Thermal conductivity (liquid) k_l $(\frac{W}{m \cdot K})$	0.0136T+1.3097, T < 1933 K<br>33.4, T ≥ 1933 K	0.091T+152.5, T < 915 K<br>90, T ≥ 915 K	0.0146T+10.3, T < 1723 K<br>26.9, T ≥ 1723 K
Solidus temperature T_s (K)	1877	873	1658
Liquidus temperature T_l (K)	1933	915	1723
Latent heat of melting L_m $(\frac{J}{kg})$	2.86×10^5	3.8×10^5	2.6×10^5
Vaporization point T_v (K)	3560	2792	3122
Latent heat of vaporization L_v $(\frac{J}{kg})$	9.255×10^6	1.053×10^7	6.336×10^6
Minimal absorptivity η_m	0.26	0.01	0.33
Molar mass M_l $(\frac{g}{mol})$	48	27	56
Surface tension σ $(\frac{N}{m})$	1.65	0.91	1.87
Thermocapillary coefficient $\frac{d\sigma}{dT}$ $(\frac{N}{m \cdot K})$	-2.6×10^{-4}	-3.5×10^{-4}	-4.9×10^{-4}
Emissivity ε	0.3	0.1	0.3
Dynamic viscosity μ_l $(Pa \cdot S)$	2.66×10^{-3}	1×10^{-3}	8×10^{-3}

Supplementary Table 1. Thermophysical properties of the employed materials (*13*).

Properties & units	Values	Properties & units	Values
**Properties of argon**		**Vaporization constants of Al6061**	
Molar mass $\boldsymbol{M_g}$ ($\frac{g}{mol}$)	39.95	Low vaporization threshold $\boldsymbol{T_L}$ ($\boldsymbol{K}$)	2800
Heat capacity (liquid) $\boldsymbol{C_{\mathrm{pg}}}$ ($\frac{J}{kg \cdot K}$)	520	High vaporization threshold $\boldsymbol{T_L}$ ($\boldsymbol{K}$)	3200
Thermal conductivity (liquid) $\boldsymbol{k_g}$ ($\frac{W}{m \cdot K}$)	1.7×10^{-2}	Ablation rate constants $\boldsymbol{a_1/b_1}$	-1.78408×10^{-6}/ 1.66819×10^{-2}
Dynamic viscosity $\boldsymbol{\mu_g}$ ($\boldsymbol{Pa\cdot S}$)	2.26×10^{-5}	Ablation rate constants $\boldsymbol{c_1/d_1}$	-5.14573×10^{1}/ 5.24582×10^{4}
**Computational constants**		Surface pressure constants $\boldsymbol{a_2/b_2}$	2.06397×10^{-4}/ -6.83622×10^{-1}
Mushy zone constant $\boldsymbol{A_{\mathrm{mush}}}$ ($\frac{kg}{m^3 \cdot s}$)	1×10^{8}	Surface pressure constants $\boldsymbol{c_2/d_2}$	-1.02617×10^{3}/ 3.80205×10^{6}
mushy zone constant $\boldsymbol{B}$	1×10^{-4}	**Vaporization constants of Ti64**	
Atmospheric pressure $\boldsymbol{P_{\mathrm{atm}}}$ ($\boldsymbol{Pa}$)	1×10^{5}	Low vaporization threshold $\boldsymbol{T_L}$ ($\boldsymbol{K}$)	3320
Gas constant $\boldsymbol{R}$ ($\frac{J}{K \cdot mol}$)	8.314	High vaporization threshold $\boldsymbol{T_L}$ ($\boldsymbol{K}$)	3920
Retro-diffusion coefficient $\boldsymbol{\beta_R}$	0.18	Ablation rate constants $\boldsymbol{a_1/b_1}$	-4.40396×10^{-7}/ 4.79205×10^{-3}
Stefan-Boltzmann constant $\boldsymbol{\sigma_{\mathrm{SB}}}$ ($\frac{kg}{s^3 \cdot K^4}$)	5.67×10^{-8}	Ablation rate constants $\boldsymbol{c_1/d_1}$	-1.72565×10^{1}/ 2.05878×10^{4}
Ambient temperature $\boldsymbol{T_\infty}$ ($\boldsymbol{K}$)	293	Surface pressure constants $\boldsymbol{a_2/b_2}$	2.98416×10^{-4}/ -3.21887
Mass diffusion coefficient $\boldsymbol{D}$ ($\frac{m^2}{s}$)	2×10^{-5}	Surface pressure constants $\boldsymbol{c_2/d_2}$	1.15055×10^{4}/ -1.35390×10^{7}

Supplementary Table 2. Properties of argon gas, computational constants, and vaporization constants of the employed materials.

Dimensionless numbers	Ti64	Al6061	SS316
Rayleigh number, Ra	6.68×10^{-2}	1.39×10^{-2}	8.45×10^{-2}
Marangoni number, Ma	1.51×10^{3}	6.83×10^{2}	1.53×10^{3}
Prandtl number, Pr	6.38×10^{-2}	1.30×10^{-2}	2.04×10^{-1}
Weber number, We	6.10×10^{3}	1.26×10^{4}	2.82×10^{3}
Bond number, Bo	2.33×10^{-4}	2.60×10^{-4}	3.61×10^{-4}
Capillary number, Ca	2.58×10^{-1}	2.39×10^{-1}	3.75×10^{-1}

Supplementary Table 3. Dimensionless numbers related to fluid flow and surface tension of the employed materials. The dimensionless numbers are approximated based on the material properties at the liquidus temperature.

Supplementary References:

1. Z. Gan, G. Yu, X. He, S. Li, Numerical simulation of thermal behavior and multicomponent mass transfer in direct laser deposition of Co-base alloy on steel. *Int. J. Heat Mass Transf.* **104**, 28-38 (2017).
2. A. A. Samokhin, Effect of laser radiation on absorbing condensed matter. *Proceedings of the Institute of General Physics Academy of Sciences of the USSR. Series Editor: AM Prokhorov*, **13**, 203 (1990).
3. D. Kondepudi, I. Prigogine, Modern thermodynamics: from heat engines to dissipative structures (John Wiley & Sons, 2014).
4. S. W. Han, J. Ahn, S. J. Na, A study on ray tracing method for CFD simulations of laser keyhole welding: progressive search method. *Weld. World* **60**, 247-258 (2016).
5. S. Pang, L. Chen, J. Zhou, Y. Yin, T. Chen, A three-dimensional sharp interface model for self-consistent keyhole and weld pool dynamics in deep penetration laser welding. *J. Phys. D* **44**, 025301 (2010).
6. J. Ye, S. A. Khairallah, A. M. Rubenchik, M. F. Crumb, G. Guss, J. Belak, M. J. Matthews, Energy Coupling Mechanisms and Scaling Behavior Associated with Laser Powder Bed Fusion Additive Manufacturing. *Adv. Eng. Mater.* **16**, 1900185 (2019).
7. Ansys Inc, ANSYS FLUENT theory guide: Release 2020 R1 (Canonsburg, PA, 2020).
8. C. W. Hirt, B. D. Nichols, Volume of fluid (VOF) method for the dynamics of free boundaries. *J. Comput. Phys.* **39**, 201-225 (1981).
9. R. Cunningham, C. Zhao, N. Parab, C. Kantzos, J. Pauza, K. Fezzaa, T. Sun, A. D. Rollett, Keyhole threshold and morphology in laser melting revealed by ultrahigh-speed x-ray imaging. *Science* **363**, 849-852 (2019).
10. W. E. King, A. T. Anderson, R. M. Ferencz, N. E. Hodge, C. Kamath, S. A. Khairallah, A. M. Rubenchik, Laser powder bed fusion additive manufacturing of metals; physics, computational, and materials challenges. *Appl. Phys. Rev.* **2**, 041304 (2015).
11. A. M. Rubenchik, W. E. King, S. S. Wu, Scaling laws for the additive manufacturing. *J. Mater. Process. Tech.* **257**, 234-243 (2018).
12. A. Arora, G. G. Roy, T. DebRoy, Unusual wavy weld pool boundary from dimensional analysis. *Scr. Mater.* **60**, 68-71 (2009).
13. J. J. Valencia, N. Q. Peter, Thermophysical properties. (2013).
