# Supplementary material for: Universal scaling laws of keyhole stability and porosity in 3D printing of metals
Source: Nat Commun. 2021 Apr 22;12:2379. doi: 10.1038/s41467-021-22704-0 (PMC8062476; doi:10.1038/s41467-021-22704-0)
Supplement: Supplementary file 3 — Description of Additional Supplementary Files [file 41467_2021_22704_MOESM3_ESM.pdf]

Description of Additional Supplementary Files

File Name: Supplementary Movie 1
Description: Progression of the melt pool and vapor depression in Al6061 bare plate under moving laser illumination. The sample thickness is 0.75 mm. The imaging frame rate is 50 000 fps. The laser spot size is 88 µm, the power is 416 W, the scan speed is 0.3 m/s. The pixel resolution of 1.98 µm. The exposure time for each image is 1 µs.

File Name: Supplementary Movie 2
Description: Progression of the melt pool and vapor depression in Al6061 bare plate under moving laser illumination. The sample thickness is 0.75 mm. The imaging frame rate is 50 000 fps. The laser spot size is 88 µm, the power is 416 W, the scan speed is 0.6 m/s. A pixel resolution of 1.98 µm. The exposure time for each image is 1 µs.

File Name: Supplementary Movie 3
Description: Progression of the melt pool and vapor depression in Al6061 bare plate under moving laser illumination. The sample thickness is 0.75 mm. The imaging frame rate is 50 000 fps. The laser spot size is 88 µm, the power is 416 W, the scan speed is 0.9 m/s. A pixel resolution of 1.98 µm. The exposure time for each image is 1 µs.

File Name: Supplementary Movie 4
Description: Progression of the melt pool and vapor depression in Al6061 bare plate under moving laser illumination. The sample thickness is 0.75 mm. The imaging frame rate is 50 000 fps. The laser spot size is 88 µm, the power is 416 W, the scan speed is 0.75 m/s. A pixel resolution of 1.98 µm. The exposure time for each image is 1 µs.

File Name: Supplementary Movie 5
Description: Progression of the melt pool and vapor depression in Al6061 bare plate under moving laser illumination. The sample thickness is 0.75 mm. The imaging frame rate is 50 000 fps. The laser spot size is 88 µm, the power is 416 W, the scan speed is 0.45 m/s. A pixel resolution of 1.98 µm. The exposure time for each image is 1 µs.

File Name: Supplementary Movie 6
Description: Progression of the melt pool and vapor depression in Al6061 bare plate under moving laser illumination. The sample thickness is 0.76 mm. The imaging frame rate is 50 000 fps. The laser spot size is 88 µm, the power is 520 W, the scan speed is 0.3 m/s. A pixel resolution of 1.98 µm. The exposure time for each image is 1 µs.

File Name: Supplementary Movie 7
Description: Progression of the melt pool and vapor depression in Al6061 bare plate under moving laser illumination. The sample thickness is 0.76 mm. The imaging frame rate is 50 000 fps. The laser spot size is 88 µm, the power is 520 W, the scan speed is 0.45 m/s. A pixel resolution of 1.98 µm. The exposure time for each image is 1 µs.

File Name: Supplementary Movie 8
Description: Progression of the melt pool and vapor depression in Al6061 bare plate under moving laser illumination. The sample thickness is 0.76 mm. The imaging frame rate is 50

000 fps. The laser spot size is 88 μm, the power is 520 W, the scan speed is 0.6 m/s. A pixel resolution of 1.98 μm. The exposure time for each image is 1 μs.

File Name: Supplementary Movie 9
Description: Progression of the melt pool and vapor depression in Al6061 bare plate under moving laser illumination. The sample thickness is 0.76 mm. The imaging frame rate is 50 000 fps. The laser spot size is 88 μm, the power is 520 W, the scan speed is 0.9 m/s. A pixel resolution of 1.98 μm. The exposure time for each image is 1 μs.

File Name: Supplementary Movie 10
Description: Progression of the melt pool and vapor depression in Al6061 bare plate under moving laser illumination. The sample thickness is 0.76 mm. The imaging frame rate is 50 000 fps. The laser spot size is 88 μm, the power is 520 W, the scan speed is 1.2 m/s. A pixel resolution of 1.98 μm. The exposure time for each image is 1 μs.

File Name: Supplementary Movie 11
Description: Progression of the melt pool and vapor depression in Al6061 bare plate under moving laser illumination. The sample thickness is 0.75 mm. The imaging frame rate is 50 000 fps. The laser spot size is 88 μm, the power is 520 W, the scan speed is 0.76 m/s. A pixel resolution of 1.98 μm. The exposure time for each image is 1 μs.

File Name: Supplementary Data 1
Description: Dataset including all process parameters, material properties, and measured keyhole dimensions.

File Name: Supplementary Data 2
Description: Dataset including transient powers with different process parameters and material properties.
